# Editorial: Exploring the framing effect on maladaptive behaviors: neural mechanisms and applications

**DOI:** 10.3389/fnbeh.2026.1816738

**Published:** 2026-03-13

**Authors:** Manuela Sellitto, Kristi R. Griffiths

**Affiliations:** 1 Department of Brain and Behavioral Sciences, University of Pavia, Pavia, Italy; 2 Cognitive Neuropsychology Centre, ASST “Grande Ospedale Metropolitano” Niguarda, Milan, Italy; 3 Milan Center for Neuroscience (NeuroMi), Milan, Italy; 4 InsideOut Institute, The University of Sydney, Camperdown, NSW, Australia

**Keywords:** addiction, amygdala, decision-making, delay discounting, framing effect, maladaptive behavior, risk-taking, vmPFC

Forty-five years ago, Tversky and Kahneman (1981) stated that “the dependence of preferences on the formulation of decision problems is a significant concern for the theory of rational choice” (p. 453). This seminal observation, based on real-life human choices, introduced the concept known as the *framing effect*: the principle that contextual features of information presentation can markedly alter choices. As a classic example, individuals may shift from risk aversion to risk seeking, and even reverse their preferences, when decisions are framed in terms of potential gains vs. losses (Berns et al., 2007), thereby challenging basic axioms of rationality (Von Neumann and Morgenstern, 1947).

Subsequent research has established that framing effects extend far beyond risk-taking to encompass areas such as future reward valuation, food preferences, and prosocial behavior, with substantial support from neuroimaging findings (Liu et al., 2020; Sun et al., 2022). For instance, during intertemporal choice, holding either reward amount or delay constant while varying the other attribute alters patience (Cao et al., 2021). These and other framing effect manipulations, targeting attention to attributes, reference dependence, time construal, and by extension, many others (Ciaramelli et al., 2019; Luo et al., 2014) have been crucial in demonstrating the malleability of intertemporal decision-making (Lempert and Phelps, 2016). Neural evidence implicates that these processes are underpinned by recruitment of regions associated with reward valuation, self-projection, self-control, and conflict monitoring, including the dorsal striatum, dorsolateral prefrontal cortex, and anterior cingulate cortex (Buckner and Carroll, 2007; Kapetaniou and Soutschek, 2025). Framing also shapes food-related decisions. Presenting taste and health information through text rather than images can reduce impulsive choice by modulating attention and engaging the conflict-monitoring network (Kruse et al., 2024; Senftleben et al., 2024). Prosocial decisions are similarly influenced; framing choices in terms of gains and losses promotes more generous choices when negative consequences of acting selfishlessly on stranger others are highlighted (Sellitto et al., 2021). Neural activity in the the ventromedial prefrontal cortex (vmPFC), the temporoparietal junction, and the insular cortex were differentially recruited during generous choices in the two frames.

Collectively, such findings highlight the potential that framing effects may have in nudging maladaptive behaviors, including addiction, gambling, and binge eating, toward more adaptive patterns at both individual and societal levels (Bickel, 2015; Diehl et al., 2018). The present Research Topic brings together conceptual and empirical contributions exploring the neural mechanisms, behavioral consequences, and applied relevance of framing effects.

What are “bad habits” exactly and what can we do about them? Wang and Monterosso examine the nature of everyday bad habits, arguing that many such behaviors (e.g., cigarette smoking) persist because the anticipated immediate gratification is overvalued relative to long-term outcomes. These behaviors typically provide something immediately appealing, but at a future cost. The authors propose that framing interventions directed at reducing delay discounting could mitigate maladaptive habits, as they could target the “temptation challenge” in addition to automaticity. Contrary to accounts portraying bad habits, such as addiction, as purely stimulus-response associations, they argue that habitual behaviors remain sensitive to changes in outcome value. Through their review of the literature, the authors identify framing strategies that can disrupt the advantages that sustain habitual behaviors. In particular, drawing on construal level theory (Trope and Liberman, 2010), they highlight strategies such as increasing the relative salience of the policy level, or bundling future choice with the immediate temptation (Ainslie and Monterosso, 2003).

What are the neuronal mechanisms implicated in the pursuit of future reward? Johnson and Grabenhorst review evidence positioning the amygdala as a central structure in future reward pursuit. From single-neuron recordings and neuroimaging studies in primates, they identify three amygdala-supported computations: (i) generating internal goals based on subjective valuation, (ii) forming behavioral plans to achieve these goals, and (iii) executing these plans and monitoring progress during multi-step decisions. As these neural processes integrate anticipated subjective reward value with costs associated with delay and effort, they are vulnerable to temporary preferences. Dynamically inconsistent valuations would lead individuals to abandon long-term goals for immediate gratification. Dysfunction in these computations may thus underlie maladaptive future-oriented behavior in psychiatric and behavioral conditions. The authors outline potential framing-based strategies based on the physiological functioning of amygdala neurons, such as emphasizing rewarding aspects of delayed outcomes while downplaying delay/effort costs, or reframing long-term goals into more proximal sub-goals to enhance patience and goal pursuit.

Is the framing effect itself susceptible to the recipient of the payoff in a gambling task? Kroker et al. investigate whether the framing effect depends on the beneficiary of the decision in a gambling paradigm. Using a classical gambling task that has sure options framed as gains or losses, combined with within-subject excitatory and inhibitory transcranial direct current stimulation (tDCS) of the vmPFC, they measured event-related fields via magnetoencephalography. They reported the expected framing effect and replicated their prior findings that participants gambled more often after inhibitory compared to excitatory vmPFC stimulation (Kroker et al., 2022). Crucially, these effects emerged only when participants made decisions for themselves rather than for another person. Neuroimaging analyses revealed spatiotemporal clusters in the prefrontal, temporal, and parietal regions aligned with the behavioral interactions. Participants also perceived self-related losses as more unpleasant, particularly under excitatory stimulation. Overall, these findings suggest that the framing effect in maladaptive behaviors stems partly from self-focused (i.e., with more concrete construal) vmPFC processing, which heightens emotional reactions to gains and losses and makes individuals more vulnerable to biased choices. Because excitatory vmPFC stimulation improved learning and reduced irrational tendencies only when decisions affected oneself, maladaptive behaviors like gambling disorder may arise from impaired self-referential valuation. Clinically, it could be useful to reduce the personal salience of risky choices, for example reframing them from a third-person or future-self perspective to increase emotional detachment. This may weaken framing biases, while neuromodulation targeting vmPFC could eventually help normalize distorted decision-making.

Does the framing effect occurs at all ages? Lacombe et al. examined developmental aspects of framing using two gambling-like tasks adapted from primate research. Despite both tasks having identical economic parameters, they elicited markedly different behaviors. When risk had to be learned through experience, both adults and children were largely risk-neutral. When probability could be inferred visually from the number of sub-options, both groups became reliably risk-prone, with particularly strong effects in children. A second study showed that providing explicit probability descriptions did not change behavior in the experience-based task, whereas reducing the visual salience of the risky option decreased risk-taking in the visually-cued task, indicating that attentional capture and framing were key drivers. Overall, this study demonstrates strong contextual dependence of risk preference in adults and children and reinforces cross-species similarities in how perceptual salience and reference framing shape decisions under uncertainty. These findings strengthen the idea that maladaptive behavior may arise not only from stable traits but from contextual cues that bias attention and shift reference points. Highly salient or visually emphasized risky options can push individuals toward risk-prone choices, especially vulnerable populations. Environments rich in attention-grabbing risky alternatives (e.g., gambling interfaces, financial apps) may amplify maladaptive behaviors. Modifying contextual framing or reducing salience could meaningfully reduce harmful decision biases.

Together, the contributions in this Research Topic illustrate how behavioral and neural insights into framing can meaningfully inform strategies for improving decision-making across diverse domains. By revealing how context, attention, and self-referential processing shape choices, this body of work highlights that maladaptive behaviors are not fixed traits but dynamic responses sensitive to subtle shifts in presentation. This has important implications for public health, clinical intervention, and policy design. Thoughtfully applied framing strategies, grounded in empirical evidence, may help guide individuals toward healthier, more future-oriented and socially beneficial decisions. Continued interdisciplinary research integrating cognitive science, neuroscience, and behavioral economics will be essential to refine these approaches and translate them into scalable, real-world interventions.
